# Isoform Sequencing and State-of-Art Applications for Unravelling Complexity of Plant Transcriptomes

**DOI:** 10.3390/genes9010043

**Published:** 2018-01-18

**Authors:** Dong An, Hieu X. Cao, Changsheng Li, Klaus Humbeck, Wenqin Wang

**Affiliations:** 1 School of Agriculture and Biology, Shanghai Jiao Tong University, 800 Dong Chuan Road, Shanghai 200240, China; dongan1983@sjtu.edu.cn (D.A.); lcslyh@yahoo.com (C.L.); 2 Institute of Biology/Plant Physiology, Martin-Luther-University of Halle-Wittenberg, Weinbergweg 10, 06120 Halle, Germany; xuan.cao@biologie.uni-halle.de (H.X.C.); klaus.humbeck@pflanzenphys.uni-halle.de (K.H.)

**Keywords:** isoform sequencing, genome annotation, novel genes, alternative splicing, long reads, single-molecule real-time sequencing, transcriptomics, plant, fusion genes

## Abstract

Single-molecule real-time (SMRT) sequencing developed by PacBio, also called third-generation sequencing (TGS), offers longer reads than the second-generation sequencing (SGS). Given its ability to obtain full-length transcripts without assembly, isoform sequencing (Iso-Seq) of transcriptomes by PacBio is advantageous for genome annotation, identification of novel genes and isoforms, as well as the discovery of long non-coding RNA (lncRNA). In addition, Iso-Seq gives access to the direct detection of alternative splicing, alternative polyadenylation (APA), gene fusion, and DNA modifications. Such applications of Iso-Seq facilitate the understanding of gene structure, post-transcriptional regulatory networks, and subsequently proteomic diversity. In this review, we summarize its applications in plant transcriptome study, specifically pointing out challenges associated with each step in the experimental design and highlight the development of bioinformatic pipelines. We aim to provide the community with an integrative overview and a comprehensive guidance to Iso-Seq, and thus to promote its applications in plant research.

## 1. Introduction

Transcriptomics is the study of the transcriptome including gene structure, expression, and regulation. Genes exhibit spatially and temporally-specific expression, which can be quantified by the second-generation sequencing (SGS) of RNA sequencing (RNA-Seq). Whereas the SGS technologies have provided massive sequence data by their high-throughput characteristic in comparison to Sanger sequencing, their application of short reads makes them poorly suited for some technical defects, including genome and transcriptome assembly, isoform detection, and methylation identification. Single-molecule real-time (SMRT) sequencing developed by PacBio offers an alternative approach to overcome these limitations and accelerate our understanding of the complexity of the transcriptome [1].

**Figure 1:**
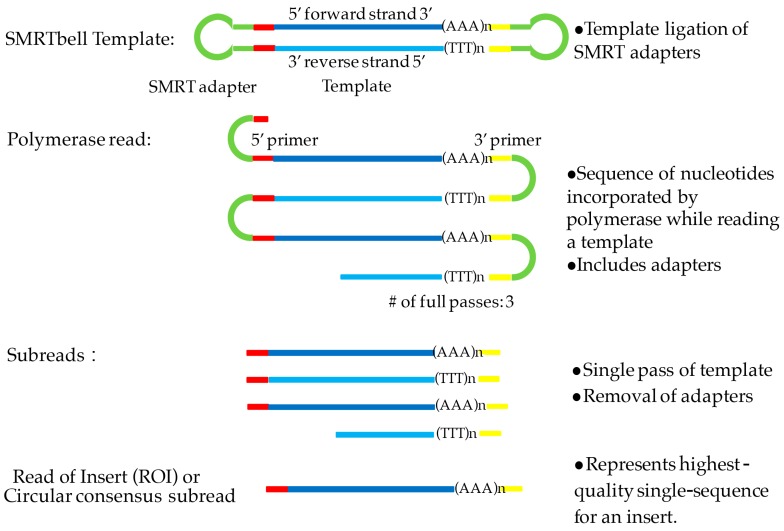
Definition of the polymerase read, subreads, and the read of insert (ROI). DNA template is labeled in blue and adapter in green. SMRT: Single-Molecule Real-Time sequencing.

PacBio sequencing captures sequences during the replication of the target DNA in real-time mode. The template includes an inserted double-stranded DNA (dsDNA) with hairpin adaptors at both ends, resulting in a closed and single-stranded circular DNA, also called a SMRTbell (Figure 1) [2]. When a library is loaded into a SMRT cell, the molecules of the SMRTbell are distributed into a sequencing unit called a zero-mode wave guide (ZMW) [3]. In each ZMW, the fixed DNA polymerase can incorporate the hairpin adaptor of the SMRTbell and start the process of replication. When the four fluorescent-labeled nucleotides are elongated with the DNA, the sensitive signals from the light pulse provide base detection [4]. The replication processes in all ZMWs of a SMRT cell are recorded by a ‘movie’ of light pulses, and the pulses corresponding to each ZMW can be interpreted to be a sequence of bases or a polymerase read. A single polymerase read can be up to 40 kb based on the library size and sequencing time. Because the SMRTbell forms a closed circle, after the polymerase replicates one strand of the target dsDNA or double-stranded complementary DNA (dscDNA), it can repeat the cycle until the reaction is terminated. However, the average length of full transcript is 1–2 kb in most plant genomes (for example, 1.6 kb in *Arabidopsis* [5], 1.8 kb in rice [6], and 1.6 kb in maize [7]), thus the long polymerase read can cover the same transcript multiple times. In this case, the polymerase read can be split into a few reads (called subreads) by the removal of adaptor sequences. The consensus sequence of multiple subreads in a single ZMW produces a read of insert (ROI) or a circular consensus sequence (CCS) read with higher accuracy (Figure 1). Accordingly, PacBio develops an independent protocol of isoform sequencing (Iso-Seq) for long-read transcriptome sequencing, including library construction, size selection, sequencing, and data processing. Iso-Seq allows direct sequencing of transcripts up to 10 kb without a reference genome. In addition, PacBio sequencing is used to detect base modifications such as methylation by taking advantage of the real-time kinetic variation interpreted from the light-pulse movie [1].

Although PacBio sequencing can generate much longer reads than SGS, the throughput is relatively low. There are 150,000 ZMWs on a single SMRT RSII cell, each of which could produce one polymerase read with an average length of 10 kb. Typically, only 35,000–70,000 of 150,000 ZMWs on one cell could successfully produce reads due to the failure of anchoring a polymerase, loading more than one template DNA or the missing of template DNA. Therefore, the typical throughput of the PacBio RS II system is about 0.5–1 Gb per SMRT cell [8]. Recently, a new system called Sequel has been developed by PacBio, producing over seven times the reads with 1,000,000 ZMWs and yielding about 3.5–7 Gb per SMRT Cell [9]. The newly developed Sequel is an ideal system for projects of the de novo assembly of plant genome and isoform sequencing of plant transcriptomes. Another problem with PacBio sequencing is its relatively high error rate (10%), whereas it can be greatly minimized by generating sufficient sequencing depth and passes in polymerase reads. In general, a coverage of 15 passes may yield > 99.3% accuracy [1,4]. However, the length of the circular consensus subread (CCS) and the number of sequencing passes are a trade-off, i.e., longer inserts yield fewer passes in a polymerase read with lower accuracy. Many hybrid sequencing strategies have been developed to make use of the accuracy of short reads and the length of PacBio reads. After correction of long reads with Illumina reads for a maize transcriptome study, the mapping rate was increased from 11.6% to 99.1% due to fixing the indel errors [10]. To examine the differential alternative splicing (AS) in various tissues, RNA-Seq data was analyzed to visualize the global AS isoforms in maize using the inbred lines B73 and Mo17, and a related species of sorghum [11]. Noticeably, the short reads could quantitate gene expression in the downstream of transcriptome analysis [12].

Here we summarize the sample preparation, library construction, and analytical pipelines of isoform sequencing (Iso-Seq). We will also discuss the applications of Iso-Seq in plant research, including identification and quantification of alternative splicing transcript isoforms, alternative polyadenylation (APA), novel transcripts, etc.

## 2. Sample Preparation and Library Construction for Isoform Sequencing 

**Figure 2:**
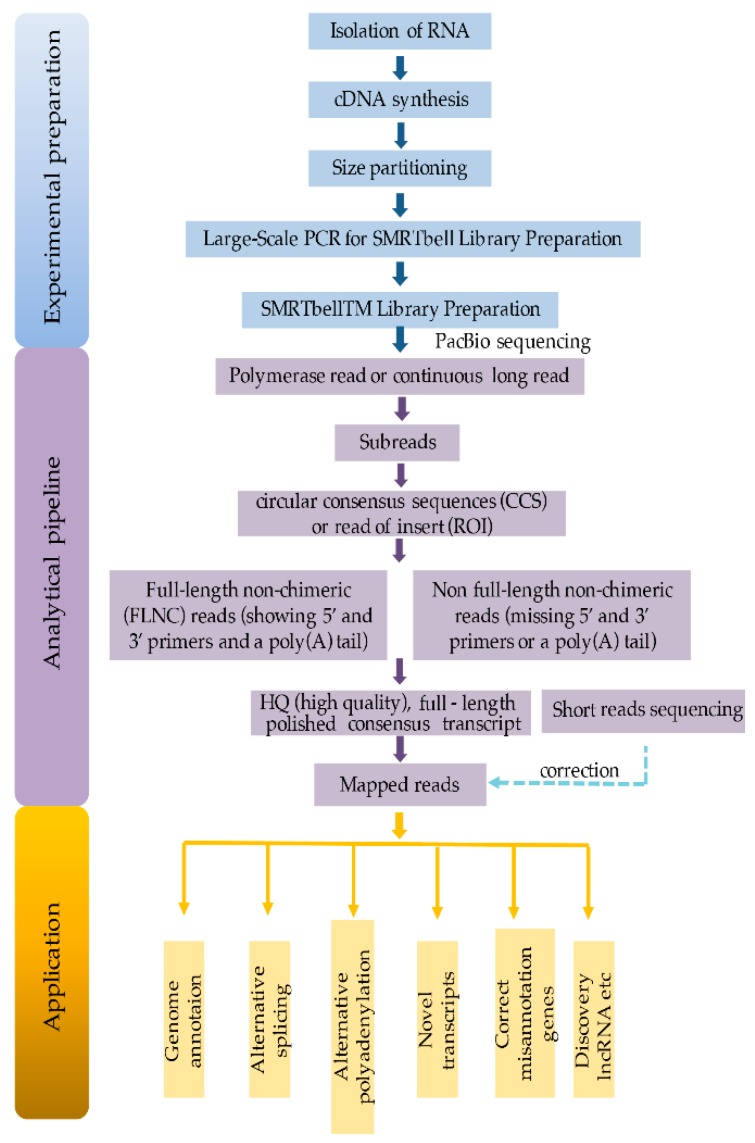
Schematic workflow of isoform sequencing.

Iso-Seq with the PacBio platform can generate full-length cDNA sequences including 5′ and 3′-UTR (untranslated region) and polyA tails of the transcripts, eliminating the transcriptome reconstruction steps. Iso-Seq provides information about alternative splicing, transcriptional start sites, and polyadenylation sites. Therefore, Iso-Seq technology has been widely used for the characterization of posttranscriptional regulatory networks. The whole workflow including experimental protocol, analytical pipelines, and application is shown in Figure 2 [3].

### 2.1. Isolation of Total RNA

The plant sample can be harvested from different tissue types (for instance, root, pollen, and embryo of maize) [13], or from certain developing stages (developing wheat grains collected at 5, 15, and 25 days after anthesis) [14]. The high quality of RNA with enough purity and integrity is critical to the success of isoform sequencing (Figure 2). The standard method of RNA extraction is via a TRIzol or Plant RNeasy kit. For example, the RNA isolations for Iso-Seq of sorghum and maize are made by TRIzol, while the samples for *Amborella trichopoda* and *Salvia miltiprrhiza* are prepared by Plant RNeasy kit (Table 1). Generally, 2–5 ug of total RNA with an RNA integrity number (RIN) greater than 8 is required. The RIN value can be decreased with the presence of abundant ribosomal chloroplast RNA even though the RNA is intact. Thus, a RIN value of 9 is considered to be high-quality RNA extracted from maize tissues including root, pollen, endosperm, embryo, and tassel, while a RIN of 8 for leaf RNA is acceptable [14,15]. A high amount and quality of RNA could reduce amplification cycles in large-scale PCR and improve the sequencing diversity. 

### 2.2. cDNA Synthesis

Isolation of polyA mRNA is required for studying the transcripts of protein-coding genes, but the Iso-Seq method is flexible, allowing different types of RNA to be sequenced (Figure 2). When the project is mainly involved with gene structure for a genome annotation, all RNAs without any filtering are subjected to sequencing. If the project aims to elucidate the regulatory network, mRNA can be selected by polyA enrichment. In most occasions, the first-strand cDNA was amplified by anchored oligo(dT)_n_ to enrich RNA with polyA tail, including mRNA and long noncoding RNA (lncRNA) for further analysis. 

An efficient method for the parallel analysis of pooled samples is to barcode each sample with unique sequences. For instance, multiplex sequencing was applied to the construction of a maize transcriptome library from six different tissues. It was found that various isoforms existed in different tissues, and the complexity of the maize transcriptome was illuminated [13]. However, barcoding samples are not always necessary because the efficient output data are reduced by the barcode sequence. An alternative way to separate samples without barcodes is to sequence the same transcriptome samples by third-generation sequencing (TGS) and SGS. The power of RNA-Seq from SGS is to quantify gene and transcript expression, whereas longer reads are capable of sequencing complete transcripts and qualifying gene features.

### 2.3. Size Partitioning

Size partitioning by the BluePippin system is the most common way to avoid an over-representation of smaller transcripts in sequencing data (Figure 2). Size selection allows for more even representation across cDNA of different size ranges, since smaller fragments may load preferentially on the sequencer. Furthermore, the second fractionation step is recommended to remove any smaller fractions from the first size selection. In general, different sizes of the cDNA libraries including <1 kb, 1–2 kb, 2–3 kb, and 3–6 kb are constructed to improve better PCR amplification, and in turn, maximally retrieve the transcript diversity and sequence (Table 1). 

### 2.4. Library Preparation

After size selection, double-stranded cDNA is not sufficient for SMRTbell library construction. PacBio recommends a PCR amplification using the KAPA HiFi Enzyme [23] with about 10 cycles (Figure 2). Then the cDNAs are transformed into a circularized molecule that called a SMRTbell template by the SMRTbell Template Prep Kit 1.0. After completing this step, the library is ready to be loaded into a SMRT Cell and subjected to sequencing on the PacBio equipment. The libraries are sequenced for 180 min movie times using the chemistry of P4C2 polymerase and 240 min movie times using P6C4 polymerase. To maximize the capture of transcript categories, RNA extractions across different tissues, developmental stages, and environmental stresses are required. In addition, sequencing depth—i.e., SMRT cell number—is also another critical factor to retrieve diverse transcripts. There is a compromise between SMRT cell numbers and the sequencing cost. In general, the Iso-Seq protocol recommends 8–50 SMRT cells to retrieve in a tissue. For reference, we summarized Iso-Seq technologies applied in plant research involved with sample collection, RNA preparation, library construction, PacBio sequencing platforms, and SMRT cell numbers (Table 1).

## 3. Bioinformatic Analysis

The raw reads generated by PacBio are usually called polymerase read or continuous long read (CLR) with an average length of 10 kb (Figure 1). Given that the average length of a transcript is 1–2 kb, a single polymerase read contains copies of the same inserts and could be split to several subreads by removing the adaptor sequences by PacBio SMRT link analysis [15]. The circular consensus sequences or ROI from multiple subreads are generated with higher accuracy by RS_IsoSeq [21] or ToFu [13,24]. When both 5′- and 3′-cDNA primers are present, as well as a polyA tail signal preceding the 3′-primer, the full-length non-chimeric read (FLNC) is defined. To improve consensus accuracy and remove the redundancy of FLNC without requiring additional sequence data, an iterative clustering for error correction (ICE) algorithm and Quiver could be performed [15] (Figure 2). 

Due to the high frequency of errors of nucleotide indels and mismatches in Iso-Seq reads, indels and mismatches are corrected via alignment with reference genomes [16]. An alternative way to overcome this limitation is to integrate short reads with long reads via hybrid sequencing, which is widely used in the global characterization of the transcriptome in plants. For example, the same RNA samples are sequenced both by PacBio and Illumina HiSeq2500, wherein the short reads are applied to verify and quantify the transcript isoforms in bamboo [15]. The splice junction of Iso-Seq isoforms is also improved with HiSeq short reads [14]. In this case, certain programs have recently been developed such as PacBioToCA (error correction via Celera Assembler) [10], LSC [25], LoRDEC [26], and proovread [27]. PacBio long reads for phage, prokaryotic, and eukaryotic genome sequencing are corrected to 99.9% accuracy by PacBioToCA [10]. The computation method of LSC applies a homopolymer compression (HC) transformation strategy to increase the sensitivity of short read against long read alignment without sacrificing alignment accuracy. For instance, LSC corrects 100,000 PacBio long reads by using 64 million short reads and reduces the error rate by more than three-fold in the transcript sequencing of human brain cerebellum [25]. The program of LoRDEC runs six times faster and requires 93% less memory than PacBioToCA and LSC, achieving a comparable accuracy [26]. The proovread program performs better than PacBioToCA and LSC by the tests of genomic and transcriptomic data from *Escherichia coli*, *Arabidopsis thaliana*, and human [27]. To give readers a broad view of sequencing depth, we summarize the numbers from isoform sequencing in terms of raw data and filtering for full-length reads (Table 2). With the current Iso-Seq workflow, ~50% of ROI could be defined as full-length ROI (containing 5′ primer, 3′ primer, and the polyA tail). More than half a million reads are the minimum number for detecting transcripts with modest abundance, while increasing the sequencing depth, will improve the chances of identifying rare isoforms and alternative splicing. Recently, the new transcriptome analysis pipelines of TAPIS (Transcriptome Analysis Pipeline for Isoform Sequencing [28]) and pipeline for Iso-Seq [29] were developed for the analysis of diploid sorghum [16] and polyploid cotton, respectively [19].

## 4. Applications in Plant Transcriptome Research

Isoform sequencing by long reads is a revolutionary technology in plant transcriptome study. The new Iso-Seq provides full transcripts up to ~10 kb from the 5′ cap to the polyA tail, avoiding read reconstruction from local information. It can retrieve most of the expressed transcripts as full-length sequences, alternative isoforms, and duplicated genes. It provides a broad spectrum of gene structure and transcriptome diversity, serving as a valuable resource to the plant research community, including:

(1) Iso-Seq can provide a reference transcriptome for the new non-model plant whose reference genome is not yet available, contributing to improved gene models and the genetic improvement in plants [20,22].
(2) Iso-Seq can generate full-length transcripts, which is fundamental to a newly sequenced genome. It provides golden evidence via alignment against genome to direct delimitate exons, splice sites, and alternative splicing junctions. The continuous sequences guarantee the better accuracy of gene annotations compared to expressed sequence tag (EST), RNA-Seq, and homology inference [30].
(3) Iso-Seq enables the improvement of the existing genome annotations or even highly characterized plant genomes such as wheat and maize that are far from complete with respect to the identification of novel genes, AS, and APA [13,14].

Here we summarize the aspects of Iso-Seq applications in terms of genome annotation, the discovery of AS and APA, and the detection of fusion genes and methylation. 

### 4.1. Genome Annotation

FLNC reads can be mapped against the reference genome using a genome mapping and alignment program (GMAP) [31]. These mapped reads can structurally and functionally annotate the genome, as well as improve genome assembly [14]. A method of validating gene models using PacBio cDNA reads has been developed, and it is shown that long reads are well suited to identify reliable gene models in de novo annotation of plant genomes. Using the training data of 2794 gene models from SMRT reads, the specificity and the accuracy of the annotation program were improved, leading to a total of 26,923 genes predicted in sugar beet [21]. Basically, most reads that could be mapped to one unique location in the genome. These reads are useful to define the gene structure and the junction of exons and introns. Transcripts that could not map to any annotated genes were defined as novel genes. Novel genes and corresponding functions could also be identified by using BLASTX against a known protein database. For instance, 2171 transcripts that were not overlapped with any annotated gene were identified as novel protein-coding genes in sorghum [16]. The missing hits of 1213 transcripts against the National Center for Biotechnology Information (NCBI) non-redundant nucleotide database and Rfam were defined to be potential novel genes in coffee. The captured longer UTRs also facilitated the identification of upstream open reading frames (ORFs) in coffee [22]. Some novel genes do not have coding potential with no more than 100 amino acids in the ORF are determined as potential lncRNA. In maize, 867 transcripts with a mean length of 1.1 kb were identified as novel high-confidence lncRNAs [13,32].

Some reads show multiple best alignments across the genome, which are most likely transcribed from highly similar homologous loci. The reads with partial mapping to two or more adjacent annotated genes indicate that these genes may be mis-annotated split genes, which are useful to improve the gene model annotation. For example, a total of 2241 genes were mis-annotated as multiple split genes, which could be merged into 1092 new loci in bamboo [15]. The remaining reads with low-quality or no significant mapping to the reference genome could be due to the missing sequences in the current draft genome that may aid future annotation of the chromosomal loci. In addition, the unmapped reads could be derived from the variety-specific genes when the mapped reference and sequenced plants are different. These variety-specific genes may also be valuable in helping genetic analysis of the traits unique in varieties. A total of 197,709 full-length non-chimeric reads were retrieved from the isoform sequencing of common wheat. The structures of 13,162 known genes were validated and 3026 novel genes were newly discovered. Additionally, 180 transcripts were found spanning two or three previously annotated adjacent loci, suggesting the contiguity of these contigs [14]. The survey of transcriptome isoform diversity by using single-molecule cDNA sequences is becoming a landmark for gene discovery and annotation in sorghum [16], maize [13], *Amborella* [17], strawberry [12], bamboo [15], and *Salvia* [18] (Table 3).

### 4.2. Alternative Splicing and Alternative Polyadenylation Discovery

**Figure 3:**
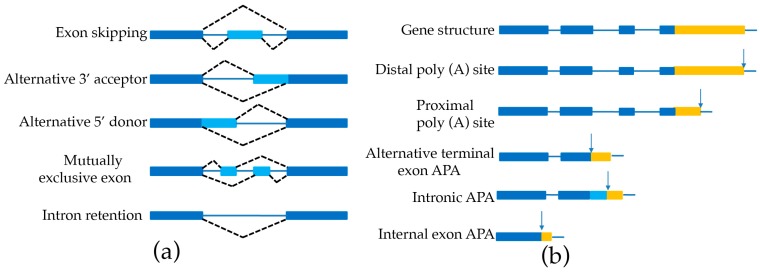
Schematic representation of alternative splicing (AS) and alternative polyadenylation (APA). (**a**) Shows alternative splicing; (**b**) shows alternative polyadenylation. The first two APA types—which both are termed as tandem 3′ untranslated region (UTR) APA—generate multiple isoforms that differ in their 3′UTR length without impacting the protein sequence encoded by the gene. The other three APA types potentially affect the coding sequences: alternative terminal exon APA, in which APA generates isoforms that differ in their last exon; intronic APA, which involves cleaving at the cryptic intronic polyA signal (PAS) with an extending terminal exon; and internal exon APA, which involves premature within one coding region with PAS. The filled dark blue boxes denote the retaining exons, and the filled light blue boxes denote the alternative exons. The blue solid lines represent introns. The black dash lines represent AS events. The filled yellow boxes represent 3′ UTR with different length, and the arrows denote PAS.

AS is prevalent in most plant genomes [33,34]. This phenomenon leads to multiple isoforms of individual genes, which dramatically increases the complexity and flexibility of the entire transcriptome and proteome. Several modes of alternative splicing are found in genomes including exon skipping (ES), alternative 3′-acceptor (AA), alternative 5′-donor (AD) mutually exclusive exon (MEE), and intron retention (IR) (Figure 3a). Several AS identifiers have been developed, such as a program to assemble spliced alignments (PASA) [17], SpliceGrapher [35], CASH [36], and the method of Astalavista et al. [37] to delimitate gene structure (Table 4). The AS event is surprisingly common in sorghum (10,053) [16], *Amborella* (4879) [17], and bamboo (21,154) [15] (Table 3).

Single-molecule long read sequencing has advantages over short read in isoform detection as long reads cover entire transcripts directly without ambiguity and re-construction from reads (Table 5). For example, the maize reference annotation identified with 2.84 isoforms per gene in comparison to 6.56 isoforms from Iso-Seq. The cotton annotation also detected more isoforms (3.93 per gene) with Iso-Seq than SGS (1.35 per gene) [38]. The reference-guided (Cufflinks) and de novo (Trinity) assembler from short reads could only reconstruct a small percentage (Cufflinks: 22%; Trinity: 8%) of PacBio isoforms in maize [13]. Meanwhile, compared with RNA-Seq data or previous annotated references, Iso-Seq retrieved a longer-length of transcripts, such as *Amborella*, maize, strawberry, and cotton (Table 5). Iso-Seq discovered more AS events (17,260) in strawberry in spite of low sequencing depth than Illumina (12,080) [12]. Deep Iso-Seq (133,229) retrieved eight times more AS events than SGS (16,437) in cotton [38]. It was found that some AS could contribute to a gain or loss of functional domains in the gene products, thus changing the modes of the fruit maturation, indicating the regulatory role of AS in plant development [39]. Furthermore, the power of Iso-Seq allowed the high efficiency of detecting fusion genes, which found 1430 members against 134 genes from SGS (Table 5).

APA of the 3′ end of mRNAs is an important co-transcriptional modification in plants [40,41,42]. Recent high-throughput studies reveal that APA increases transcriptome complexity by generating transcript isoforms that differ in the coding region or 3′ UTR, as shown in Figure 3b, thereby regulating mRNA transportation, localization, stability, and translation [43,44]. However, it is not trivial to identify APA with the conventional SGS approach due to its short-reads. A recent study by using polyadenylation site sequencing (PAS-Seq) with SMRT technology characterized 6311 genes containing APA sites in bamboo [15]. In another study in sorghum, 7700 genes were found to have two or more polyadenylation sites with Iso-Seq reads [16], indicating that APA could be accurately annotated in plants by PacBio sequencing. 

Biotic and abiotic stresses dramatically affect post-transcriptional regulation through AS and APA in plants [40]. The well-known examples are related to flowering time control pathways [45] and stress responses [46]. Iso-Seq for plant samples with the stress treatments will allow genome-wide uncovering of the stress-regulated isoforms. Such information would be critical in addressing the role of stress-regulated post-transcriptional splicing and polyadenylation in the adaptation to various stresses for plants [16]. Meanwhile, AS and APA show tissue- or developmental-specific expression patterns. For instance, from Iso-Seq data, the maize *CSR1* gene was discovered to have two novel isoforms: one in the root and the other in the tassel [13]. Another example was cellulose synthase gene (*CesA*) in bamboo involved in the formation of cell wall, which was identified to be regulated by APA [15]. 

Quantification by short read transcriptome sequencing from different plant stages and stresses will further discover the biological functions of AS and APA events [47], whereas Iso-Seq data could also be used to measure the gene expression level with counting full-length transcripts at whole transcriptional level. GFOLD, which is specifically designed for data without biological replicates is used to search for differentially expressed genes between different samples [48]. Using GFOLD, 186 differentially expressed genes with a three-fold cutoff have been identified in sorghum [16]. The program of SQANTI could also extensively quantify gene expression with long read transcript sequences [49].

### 4.3. Fusion Genes Determination

A fusion transcript is a chimeric RNA encoded by a fusion gene or by two different genes by subsequent trans-splicing. To determine fusion transcripts, the criteria used for a single transcript were as follows: firstly, full-length transcripts map to two or more loci in the genome and each mapped locus must be at least 100 kb apart; secondly, each mapped locus must align with at least 10% of the transcript and the combined alignment coverage must be at least 99% [13]. The isoform detection and prediction tool (IDP) has also been recently released for the identification of fusion genes, fusion sites, and fusion gene isoforms from cancer transcriptomes [50]. Obviously, RNA-Seq has a great limitation in discovering the fusion genes due to its mapping uncertainty from the short reads or assembly problem, whereas Iso-Seq can obtain the full-length transcripts immediately showing the priority to detect fusion genes. However, short reads generated from the SGS platform could be used to validate candidate fusion transcripts. For example, 1430 fusion transcripts were identified by TGS in maize, of which 134 fusions were validated using an Illumina pair-end read approach (Table 5). An important observation is that fusion events were more likely to occur inter-chromosomally than intra-chromosomally and tended to occur near chromosome termini [13]. 

### 4.4. Methylation Detection

DNA modifications affect a variety of processes including DNA replication, repair, and transcription regulation [55]. Cytosine methylation is the most well-studied DNA modification. Bisulfite sequencing—the most common high throughput sequencing method for genome-wide detection of these epigenetic events—typically uses Illumina short reads to discriminate methylated cytosine from unmethylated ones with the conversion of cytosine to uracil, but it requires a well-defined reference genome and is unable to recognize different types of cytosine methylation, such as C, m5C, and 5hmC [56,57].

In contrast to the conventional bisulfate sequencing, PacBio sequencing uses an alternative approach to directly detect many native epigenetic modifications [58]. It monitors the time between base incorporations in the read strand, called inter-pulse durations (IPDs). The difference of IPDs between normal and modified bases serves as a signal to detect base modifications. Utilizing this character, Wang et al. investigate the relationship between methylation and alternative splicing in maize [13], and another study found that CG methylation exhibited differences in isoforms during fiber development in cotton [19]. 

## 5. Conclusions

Recent advances in short and long read sequencing technology have led to the dramatic increase of sequenced plant genomes. Given that the assembled genome simply serves as a reference, more attention is paid to identifying the genomic features that contribute to phenotypic traits and deciphering their biological significance. The major challenge in biology is to understand how the same genome can produce different tissue types and how gene expression is regulated. The plant transcriptome is extremely complex, showing the spatial and temporal-specific pattern in terms of tissues, circadian clock, and various stresses. Studies have provided valuable insights regarding gene differential expression, gene splicing, and posttranscriptional modifications by RNA sequencing using next-generation sequencing. However, isoform identification is still a limiting factor for RNA-Seq experiments. Iso-Seq shows the revolutionary accomplishment, providing comprehensive transcriptome resource including AS, APA, gene fusion, methylation of DNA, and transcription start site due to the generation of high-quality full-length isoforms. Still, Iso-Seq struggles with a substantial sequencing error (~10%) due to small indels. It has been shown that error correction of Iso-Seq by using RNA-Seq data provides more accurate mapping in maize [10]. Thus, a hybrid approach that combines isoform sequencing with full-length transcripts and RNA-Seq capable of fixing sequence error and quantifying gene expression is the optimal solution to study plant transcriptomes. 

## Figures and Tables

**Table 1:** Summary of sample collection, RNA extraction, library construction, sequencing platform, and throughput for isoform sequencing in plants.

Species	Sample Collection	RNA Extraction	Size-Fractionated Libraries	Platform and Throughput	Ref.
sorghum (BTx623)	Control and drought treatment of 7-day-old seedlings for 6 h	TRIzol reagent (Invitrogen, Carlsbad, CA, USA) with DNaseI (Fermentas, Waltham, MA, USA)	1–2 kb and 2–6 kb	PacBio RS II with 28 SMRT cells	[16]
maize B73	Root, pollen, embryo, endosperm, immature ear and immature tassel	TRIzol reagent (Invitrogen, Carlsbad, CA, USA) with RQ1 DNase (Promega, Madison, WI, USA)	<1 kb, 1–2 kb, 2–3 kb, 3–5 kb, 4–6 kb and >5 kb	PacBio RS II with 47 SMRT cells	[13]
wheat Xiaoyan	Unfertilized caryopses and developing grains	RNA extraction kit (Takara Biotechnology, Dalian, Liaoning, China) with TURBO DNaseI (Promega, Madison, WI, USA)	<2 kb, ≥2 kb	PacBio RS II with 8 SMRT cells	[14]
*Amborella trichopoda*	Young leaves and female flowers	CTAB method and RNeasy Mini extraction kit (Qiagen, Hilden, Germany) with TURBO DNA-free Kit	1–2 kb, 2–3 kb and >3 kb	PacBio RS II with 19 SMRT cells	[17]
wild strawberry	Receptacle of five different stages	Plant Total RNA Isolation Kit (Sangon Biotech, Shanghai, China)	1–2 kb, 2–3 kb and >3 kb	PacBio RS with 13 SMRT cells	[12]
moso bamboo	Underground rhizome, lateral rhizome, shoot, root, and leaf	RNAprep Pure Plant Kit (Tiangen, Beijing, China) with DNase I	1–2 kb, 2–3 kb and >3 kb	PacBio RS II with 7 SMRT cells	[15]
*Salvia miltiorrhiza*	Periderm, phloem, and xylem from roots	RNeasy Plus Mini Kit (#74134, Qiagen, Hilden, Germany)	<1 kb, 1–2 kb, 2–3 kb and >3 kb	PacBio RS with 8 SMRT cells	[18]
cotton	Root, hypocotyl, leaf, petal, anther, stigma; fibre samples	Spectrum Plant Total RNA kit (Sigma-Aldrich, St. Louis, MI, USA)	1–2 kb, 2–3 kb and 3–6 kb	PacBio RS II with 30 SMRT cells	[19]
sugarcane	Leaf, internode, and root tissues of different stages	TRIzol (Invitrogen) and Qiagen RNeasy Plant minikit (#74134, Qiagen, Hilden, Germany)	0.5–2.5 kb, 2–3.5 kb, 3–6 kb and 5–10 kb	PacBio RS II with 6 SMRT cells	[20]
sugar beet	Seedlings	Nucleospin Plant RNA kit (Macherey-Nagel, Duren, Germany)	1–2 kb, 2–3 kb and >3 kb	PacBio RS with 6 SMRT cells	[21]
coffee bean	Immature, intermediated, and mature fruits	TRIzol plus RNA purification kit (Invitrogen, Carlsbad, CA, USA), the RNeasy Plant Mini Kit (#74903, Qiagen, Hilden, Germany)	0.5–2.5 kb, 2–3.5 kb, 3–6 kb and 5–10 kb	PacBio RS II with 2 SMRT cells	[22]

**Table 2:** Sequencing depth in Iso-Seq.

Species	ROI	Full-Length ROI	Error Correction FLNC Reads	Mapped Reads
sorghum (BTx623)	1,838,330	884,638	NA	867,089
maize B73	3,716,604	1,553,692	643,330	606,145
wheat Xiaoyan	240,312	NA	197,709	91,881
*Amborella trichopoda*	660,458	217,954	146,686 ^1^	124,509 ^2^
wild strawberry	442,601	354,393	85,416	82,360
moso bamboo	288,312	147,362	146,225	145,522
*Salvia miltiorrhiza*	796,011	223,368	NS	NA
cotton	2,542,318	1,096,932	NA	339,230
sugar cane	290,393	186,999	107,604	74,716
sugar beet	395,038	109,920	NA	107,721
coffee bean	433,877	233,464	NA	NA

ROI: read of insert; FLNC reads: full-length non-chimeric reads; NA means the data which are not presented in the literature; ^1^ denotes reads corrected by ICE-Quiver; ^2^ denotes mapped reads obtained by two full-passes full-length non-chimeric read of insert (flncROIs) data; NS means the detail numbers are not shown although the analysis has been done.

**Table 3:** Plant genome annotation by using Iso-Seq.

Species	Isoform	Novel Transcripts	AS	APA	Novel Genes	lncRNA	Mis-Annotated Genes
sorghum (BTx623)	27,860	11,342	10,053	11,013	2171	540	941
maize B73	111,151	65,350	NS	NA	2253	867	2199 *
wheat Xiaoyan	22,768	9591	NS	NA	3026	NA	180
*Amborella trichopoda*	10,617	3680	4879	NA	510	NA	3255
wild strawberry	33,236	5501	17,260	NA	3649	NA	NA
moso bamboo	42,280	35,447	21,154	6311	8091	3096	2241
*Salvia miltiorrhiza*	160,468	NA	4165	NA	NA	11,046	NA
cotton	176,849	13,551	133,329	43,784	NA	2447	NA
sugar cane	107,598	2450	4870	NA	NA	2426	NA
sugar beet	NA	NA	NA	NA	NA	NA	4000
coffee bean	95,995	NA	NS	NS	1213	NA	NA

AS, alternative splicing; APA, alternative polyadenylation; lncRNA, long non-coding RNA; NA means the data is not available, and NS means detail numbers are not shown although the analysis has been done; * There were 2199 transcripts from Iso-Seq data covering more than one annotated V3 gene. It was confirmed that 682 (81%) out of 844 Gramene gene models were mis-annotated, while the remaining genes need further evidence to support whether they were mis-annotated.

**Table 4:** Bioinformatic programs applied in plant Iso-Seq analysis.

Species	Read Processing	Correction	Mapping	AS	Novel Gene	APA
sorghum (BTx623)	TAPIS	LoRDEC, proovread and TAPIS	GMAP	SpliceGrapher	TAPIS	TAPIS
maize B73	ToFU	ICE-Quiver	GMAP	AStalavista	BLASTN	NA
wheat Xiaoyan	SMRT analysis	SMRT analysis, proovread	GMAP	In-house perl script	GMAP	NA
*Amborella trichopoda*	SMRT analysis_v2.2.0	minFullPasses, LSC-corrected and ICE-Quiver	GMAP, BLAT	PASA, de novo AS detection	NA	NA
wild strawberry	RS_IsoSeq_v2.3	ICE-Quiver, LoRDEC	GMAP	AStalavista	NA	NA
moso bamboo	SMRT analysis_2.3.0	LSC	GMAP	AStalavista	TAPIS	TAPIS
*Salvia miltiorrhiza*	SMRT analysis_2.2.0	LSC	GMAP	SPLICEMAP	SPLICEMAP	NA
cotton	SMRT analysis	pipeline-for-Iso-Seq	GMAP	alternative_splice.py	BLAST	SMRT analysis
sugar cane	SMRT analysis_2.3.0	ICE-Quiver, proovread, and LoRDEC	GMAP	TAPIS	BLAST	NA
sugar beet	SMRT analysis_v2.0	Proovread, normalize-by-median.py	GMAP, AUGUSTUS	NA	NA	NA
coffee bean	RS_IsoSeq_v2.3	ICE-Quiver	BLAST	BLAST	BLAST	BLAST

GMAP, genome mapping and alignment program; ICE, iterative clustering for error correction; PASA, program to assemble spliced alignments; IDP, isoform detection and prediction tool; TAPIS, transcriptome analysis pipeline for isoform sequencing; SMRT, single-molecule real-time. alternative_splice.py [29]. NA means the data is not available.

**Table 5:** Comparison of detecting efficiency in terms of isoform number, average gene length, AS events, and fusion genes between Iso-Seq and SGS.

	Species	Iso-Seq	SGS/Sanger	Reference
Isoform number per gene	cotton	3.93	1.35	[19,51]
	maize B73	6.56 *	2.84 *	[13]
Total isoform number	wild strawberry	26,676	20,705	[12]
	moso bamboo	42,280	10,471	[15]
Average gene length (bp)	*Amborella trichopoda*	2044	950 ^1^	[17]
	maize B73	2632	1684	[7,13]
	wild strawberry	2466	1187	[12,52]
	cotton	2175	1462	[19,51]
AS events	wild strawberry	17,260	12,080	[12]
	cotton	133,229	16,437	[19,53]
Number of fusion genes	maize B73	1430	134	[13]

**^1^** denotes data downloaded from the *Amborella* Genome database [54]. * The PacBio long read data identified 15,146 genes with an average of 6.56 isoforms, more than twice the number of the maize V3 annotation from SGS/Sanger data [11]. The PacBio Iso-Seq data has been included in the current version of annotation, which greatly improved existing gene models although only ~70% of the total maize genes were captured [11].
